# Impact of the implementation of a telemedicine program on patients diagnosed with asthma

**DOI:** 10.1186/s12890-024-02843-y

**Published:** 2024-01-13

**Authors:** Héctor Cabrerizo-Carreño, Mariana Muñoz-Esquerre, Salud Santos Pérez, Ana Maria Romero-Ortiz, Núria Fabrellas, Eva Maria Guix-Comellas

**Affiliations:** 1 Bellvitge University Hospital, Department of Pulmonary Medicine, L’Hospitalet de Llobregat, Catalunya, ES Spain; 2 Department of Fundamental and Medico-Surgical Nursing, Nursing School, Faculty of Medicine and Health Sciences, University of Barcelona, Barcelona, Catalunya, ES Spain; 3 Pneumology Research Group, Bellvitge Biomedical Research Institute (IDIBELL), L’Hospitalet de Llobregat, Catalunya, ES Spain; 4 Department of Public Health, Faculty of Medicine and Health Sciences, School of Nursing, University of Barcelona, Barcelona, Catalunya, ES Spain; 5 Nursing Research Group, Bellvitge Biomedical Research Institute (IDIBELL), L’Hospitalet de Llobregat, Catalunya, ES Spain; 6 Facultat de Medicina i Ciències de la Salut, University of Barcelona, L’Hospitalet de Llobregat, Catalunya, ES Spain

**Keywords:** Asthma, Nursing, Telemedicine, Asthma control, Telemonitoring, Adherence to therapy, Quality of life

## Abstract

**Background:**

Asthma is one of the most common respiratory ailments worldwide. Despite broad understanding of the illness and of the available therapeutic options for it, patients with serious asthma suffer poor monitoring of their illness in 50% of cases.

**Aim:**

To assess the impact of the implementation of a mobile application (ESTOI) to control asthma in patients diagnosed with the illness, their adherence to treatment, and their perceived quality of life.

**Methodology:**

Randomized clinical trial with 52 weeks’ follow-up of patients with asthma seen in a specialized hospital for their treatment in Spain. Some 108 included patients will be divided into two groups. The intervention group will undergo more exhaustive follow-up than normal, including access to the ESTOI application, which will have various categories of attention: control of symptoms, health recommendations, current treatment and personalized action plan, PEF record, nutritional plan, and chat access with a medical team. The asthma control questionnaire ACT is the main assessment variable. Other variables to be studied include an adherence test for the use of inhalers (TAI), the number of exacerbations, maximum exhalation flow, exhaled nitric oxide test, hospital anxiety and depression scale, asthma quality-of-life questionnaire, forced spirometry parameters (FVC, FEV1, and PBD), and analytic parameters (eosinophilia and IGE). The data will be collected during outpatient visits.

**Trial registration:**

This trial has registered at ClinicalTrials.gov (Identifier: NCT06116292).

## Background

Asthma is one of the most common respiratory ailments worldwide [1]. It was estimated in 2019 that asthma affected 262 million people and caused 461,000 deaths [2]. In recent decades there has been a significant increase in the prevalence of asthma worldwide. According to the *Global Burden of Disease* study in 2015, between 1990 and 2015 the prevalence of the illness increased by 12% [3].

Asthma is manifested by bronchial hyper-response and variable obstruction of the air flow, which may be partially or totally reversible [1]. Its main symptoms are cough, wheezing, shortness of breath, pressure in the chest, and a limitation in the flow of expired air [4]. As it is a chronic illness, the aim in treatment is to reach and maintain control of the pathology and prevent future risk, especially of exacerbation, which may be life-threatening to the patient [1, 4].

Despite widespread knowledge of the illness and of the available therapeutic options, asthmatic patients show poor control of the illness in 50% of cases [5]. Because of this, symptoms may be persistent and may include constant exacerbations that present a great future risk [6, 7]. In part, poor control of the illness may be due to a lack of adherence and therapeutic compliance, to environmental exposures, and to poor control of comorbidities that directly affect asthma such as obesity, chronic sinusitis, allergic rhinitis, gastro-esophageal reflux, anxiety, and depression [1, 8].

In recent years there has been an increase in the demand for telemedicine in combination with or as an alternative to face-to-face clinical care [1]. This has led to the creation and management of new models of patient care [4, 9]. Follow-up and monitoring control of patients with asthma in accordance with evidence-based recommendations must include in assessment a monitoring of symptoms and acute incidents, continuous assessment of causative agents and comorbidities that have an impact on control of the illness, changes in pulmonary function, the side effects of treatment, and, finally, the ability of patients to carry out personalized and predesigned action plans [1, 4, 9].

Recent studies have highlighted the great potential of mobile applications to improve self-care of patients with asthma, as an efficient way to offer and share information, in addition to being of low cost and easily accessible to the population [10–12]. Despite this, owing to the diversity of interventions and their results in recent years, there is a lack of consensus regarding the type of intervention best suited to asthmatic patients and the tools needed to improve control of their illness [10–15].

For this reason, the present project has set out to develop and use a telemedicine tool for global management of patients with asthma, in order to improve adherence to treatment, increase control of the illness, empower the patients, personalize their care, and increase the efficiency of the circuit, thereby improving the quality of care.

## Aim

The aim of the present study is to assess the impact of the implementation of a mobile application (ESTOI) to control asthma in patients diagnosed with the illness, their adherence to treatment, and their perceived quality of life.

## Method

### Design

A simple-blind randomized clinical trial will be carried out, with 52 weeks of follow-up of patients with asthma. The study will have two arms: the control cohort will receive normal care, while the intervention group will additionally have access to the mobile application (ESTOI).

### Setting and subjects

The study will be carried out in the pneumology service of the Bellvitge University Hospital in Barcelona, during the period 2023–2025. The participants will be asthmatic patients seen for the first time in the hospital out-patient clinic who fulfill the following selection criteria (Table 1).

**Table 1:** Key inclusion, exclusion, and withdrawal criteria

Key inclusion criteria	
• Age ≥ 18 years with a diagnosis of asthma based on GEMA 5.2, 2022 [1] • Patients seen in the pulmonology service of the center. • Patients who have not previously received asthma education. • Capable of giving signed informed consent.	
Key exclusion criteria	
• Patient does not have a mobile device with Android or IOS system. • Lack of minimum technological knowledge for the use of the application (ESTOI). • People participating or have participated in a clinical trial in the last 6 months. • Patients diagnosed with other respiratory diseases except for obstructive sleep apnea (OSA), Asthma-COPD overlap syndrome (ACOS). • Patient with palliative or severe chronic illness that limits life expectancy.	
Key withdrawal criteria	
• Voluntary withdrawal from the study by the patient. • Lost to follow-up. • Death of the patient.	

### Calculation of the sample

In order to calculate the sample size, a screening of 60 patients seen in the pneumology unit of the center was made with the ACT questionnaire, in order to learn the degree of current control of the asthma of those patients.

With a CI of 95% and a standard deviation of 4.95, the average ACT score of the patients seen in our center was 18.43 ± 1.25. The calculation of the sample size was based upon the established aims. We used the Granmo 7.12 program for two independent averages, accepting an alpha risk of 0.05 and a beta risk of 0.2 in bilateral contrast. Some 54 subjects were determined to be needed for each group in order to detect a difference equal to or greater than 3 units in the ACT questionnaire [16, 17]. We assumed a common standard deviation of 4.95 (obtained by screening in our center) and calculated a rate of loss in follow-up of 20%.

### Sampling technique

The patients will be included in the study by means of accidental probabilistic sampling. Assignment of the intervention to be carried out will be randomly made, according to a randomization grid designed using the software of random.org [18].

### Variables

The main variable of the study will be ACT. Evaluation will be made of the participants in both groups, at the beginning of follow-up, at 6 months, and at 12 months. The results obtained will be compared. In addition, sociodemographic variables will be collected, such as age, sex, education level, and experience in the use of mobile applications. The study variables are detailed in Table 2.

**Table 2:** Study variables

Variable	Description	When will it be used?
**Asthma Control Test (ACT)**	ACT is a 5-item questionnaire that assesses the level of control of asthma symptoms during the previous 4 weeks [19].	CG at each visit (v1, v2, v3) IG once a month (using the ESTOI app) and at each visit of the study (V1, V2, V3)
**Test of the Adherence to Inhalers (TAI)**	TAI is a 12-item questionnaire that assesses adherence to inhalers for patients with Asthma or COPD [20].	CG at each visit (v1, v2, v3) IG once a month (using the ESTOI app) and at each visit of the study (V1, V2, V3)
**Asthma Quality of Life Questionnaire (AQLQ)**	AQLQ is a 32-item questionnaire that assesses the quality of life of patients with asthma. Covers 4 dimensions (breathlessness, mood, social limitation and worrying) [21].	Both groups (IG and CG) at each visit (V1, V2, V3)
**The Hospital Anxiety and Depression Scale (HADS)**	HADS is a questionnaire for detecting affective disorders in hospital settings with outpatients. Is frequently used to evaluate populations with chronic diseases [22].	Both groups (IG and CG) at each visit (V1, V2, V3)
**Blood eosinophil count**	Eosinophils are actively involved in diseases caused by parasites and in allergic reactions. In the case of asthma, having eosinophils in range is usually an indicator of good control [4].	Both groups (IG and CG) at V1 and V3
**Immunoglobulin E (IgE)**	IgE is an antibody involved in airway inflammation and allergic reactions. It plays an essential role in modulating severity as a direct association between IgE concentrations and increased disease severity, bronchial hyperresponsiveness and reduced lung performance [4].	Both groups (IG and CG) at V1 and V3
**Exacerbation**	In asthma an exacerbation is considered a worsening of asthma symptoms that requires medical intervention and has at least 1 of the following 3 elements listed below for at least 2 consecutive days: Worsening of asthma signs/symptoms (dyspnea, wheezing, nocturnal awakenings, or chest tightness), increased use of rescue medication or deterioration of lung function [1].	CG at each visit (v1, v2, v3) IG once a month (using the ESTOI app) and at each visit of the study (V1, V2, V3)
**Peak Expiratory Flow (PEF)**	PEF is the highest airflow achieved in a forced expiration after an also forced inspiration, measured using a Peak Flow Meter. It is used as a predictor of airway obstruction [23].	Both groups (IG and CG) once a week (using the ESTOI app in case of IG) and at each visit of the study (V1, V2, V3)
**Forced vital capacity (FVC)**	FVC is the total volume of air that can be exhaled during a maximum effort of forced expiration without a time limit [24].	Both groups (IG and CG) at each visit (V1, V2, V3)
**Forced expiratory volume in one second (FEV1)**	FEV1 is the volume of air exhaled in the first second under force after a maximal inhalation [24].	Both groups (IG and CG) at each visit (V1, V2, V3)
**Bronchodilator Responsiveness (BDR)**	BDR determines the degree of airflow improvement after the administration of a Beta-2 adrenergic agonist. The test is considered positive when there is an increase from baseline values of FEV1 by at least 12% and 200 mL [24].	Both groups (IG and CG) at each visit (V1, V2, V3)
**Fractional exhaled nitric oxide (FeNO)**	FeNO is a useful and noninvasive biomarker for eosinophilic airway inflammation, particularly in asthma [25].	Both groups (IG and CG) at each visit (V1, V2, V3)

*CG* Control group, *IG* Intervention group

### Procedure and instruments

**Fig. 1:**
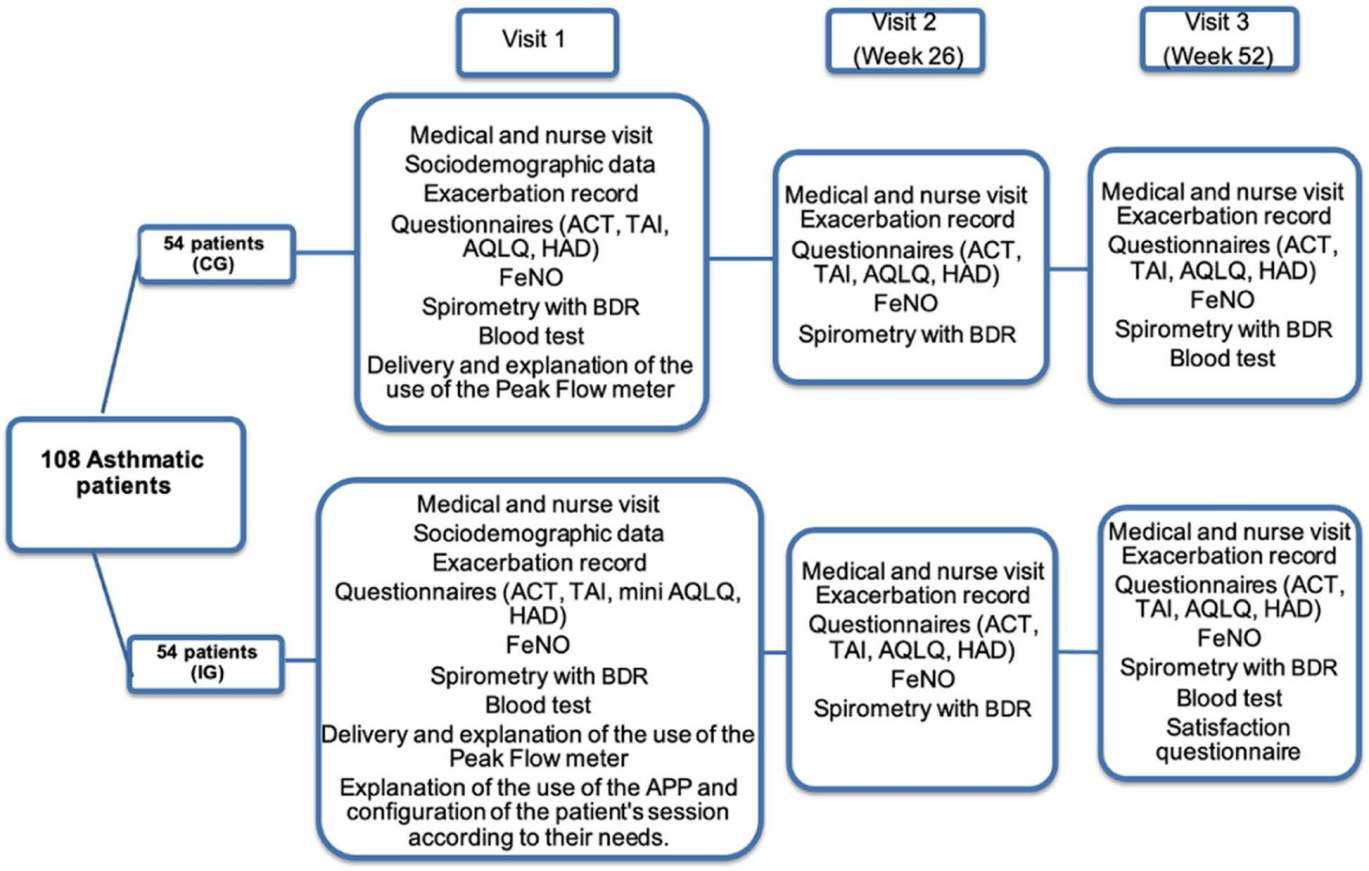
Flow chart CG: Control group; IG: Intervention group; BDR: Bronchodilator responsiveness

The project will be structured in three visits (Fig. 1, Flow chart).

At the first visit, all of the participants will be seen by the out-patient pneumology unit for classification of their asthma, renewal of their inhalers, and preparation of orders for complementary tests. Following this, the control group will receive instructions provided by a nurse specialized in asthma concerning the illness, the treatment to follow, signs to watch out for, plan of action, and a review of the triggers of the illness. Meanwhile, the patients from the intervention group, in addition to this instruction, will also be included in the database of the ESTOI application and will be provided access to the patient homepage, where they may find all the information needed for the management of their asthma. Both groups will be provided with a Peak Flow device and will receive instruction on how to use it and record the results in a diary.

At the conclusion of the first visit, all of the participants will be given an appointment for a second visit as well as a contact phone number to resolve any questions that may arise.

At the conclusion of the third visit, the intervention group will complete a questionnaire to determine their satisfaction with the app that they used.

All of the relevant data from the study will be collected in the case report form of each patient for subsequent analysis. The case report forms will be housed in the medical center in a locked room to which only the research team will have access.

At each of the three visits in the study, the nurse specialized in asthma will be responsible for compiling the study questionnaires (ACT, TAI, AQLQ, and HADS) of all the patients, reviewing the records of withdrawal from medication, carrying out a FeNO test, doing spirometry with bronchodilator, ordering bloodwork, and making on-site PEF measurement. All of the data will be duly recorded in the case report forms.

A nurse trained in telemedicine will be in charge of the management and monitoring of the patients using the application. The nurse will also be responsible for making appointments, reviewing the clinical records of all the patients included in the study, and managing the case report and informed consent forms. The nurse specialized in asthma will be responsible for administering the questionnaires, collecting the case report form data, and the carrying out of the blood tests and pulmonary function testing.

### ESTOI application for the management of asthmatic patients

The mobile application ESTOI, created and managed by the research team of this study, is designed to provide, in a personalized manner, all of the tools normally used in a hospital for the management and control of asthma. ESTOI has a number of components that may be managed and modified by the researchers in response to the needs of each patient. It is available for both Android and IOS devices.

In the first component, ‘CONTROL OF SYMPTOMS’, the participants respond to questions about their health that are designed in a personalized manner in order to learn of their current asthma state. Each month the ACT, TAI, number of exacerbations, and weight will be recorded, as well as, depending on the case, data on the control of symptoms of rhinitis, nasal polyposis, RGE, allergies, and smoking habit, if any. Depending on the results obtained from the questionnaire, the patient will be given recommendations or alerts in accordance with the seriousness of their situation, and the application will automatically alert the medical team when a serious worsening in control of the illness is detected, so as to allow for management of the situation and avoidance of future risk.

In the second component, ‘HEALTH RECOMMENDATIONS’, all of the information that the patient needs to manage the pathology is shown, including how to control it and what to do in the event of a worsening of the symptoms. The recommendations that appear in this component will depend on the patient’s state and comorbidities; only information appropriate to the current state of the patient will be available to him or her.

In the third component, ‘YOUR TREATMENT’, appears up-to-date, personalized information concerning current medication used by the patient to treat asthma, the dosage and times to take the medication, and, in the case of inhalers and biological therapy, a video explaining how they are to be administered. This component also includes the plan of action, showing the medication to be taken by the patient in the event of a worsening of the asthma, as well as in the event of an emergency. Both the treatment and the plan of action will be reviewed and modified, if necessary, at each patient visit to pneumology.

In the fourth component, ‘PEAK FLOW’, the patient can record the measurements made using the Peak Flow Meter at home. The peak flow is calculated automatically in order to reveal whether there have been significant changes in the values for pulmonary function, so that treatment can be modified and adjusted to current needs without the need for the patient to visit a medical center.

The fifth component, ‘NUTRITIONAL PLAN’, provides a guide to daily foods so that the individual may adapt the tools and advice to his or her needs.

The sixth and final component, ‘MESSAGING’, provides for two-way communication between the participants and the members of the specialized asthma medical team, allowing for additional information to be provided and for questions to be answered.

### Analysis of the data

The continuous variables are expressed as mean and standard deviation in case of normal distribution, and median and interquartile range in case of non-normal distribution. The qualitative variables are to be defined as frequencies and percentages. For comparison of the continuous variables the variance analysis method (ANOVA) or the Kruskal-Wallis test will be used. For analysis of the main assessment, the Chi-squared test will be used. Stepwise logistic regression models will be used to select the subgroup with parameters significantly associated with poor control of asthma. A priori, the factors to be included in the analysis are age, number of years suffering asthma, education level, IMC, smoking, environmental exposures, pre-BD FEV1, post-BD FEV1, FeNO, blood eosinophils, general and specific blood IgE, ACT, TAI, HAD, AQLQ, number of exacerbations, and control of comorbidities that affect asthma (GERD, rhinitis, nasal polyposis, overweight/obesity, and smoking). Statistical significance will be set at *p* < 0.05.

## Discussion

The present study represents an attempt to improve control of the illness, the quality of life of the patients at our center, and their adherence to the treatment for asthma, using telemedicine as the main tool, in combination with normal care. There is scientific evidence that carrying out more continuous follow-up of patients increases their knowledge of the pathology and of how to monitor it, improves communication between patient and professional, and personalizes resources in accordance with the needs of the individual, thereby empowering the patient to achieve greater control of his or her asthma [1, 10, 11]. It has also been demonstrated that keeping the main comorbidities (allergies, obesity, rhinitis, nasal polyposis, GERD, and stress) under control is essential to improving the health of the patient with asthma [1, 4].

There is at present a varied typology of interventions based on telemedicine to achieve improved management of the illness. The most frequently used, in terms of channel of communication, are online chats and meetings [26, 27], mobile applications [28–32], SMS [33, 34], and websites [34–36]. In terms of content, they are remote monitoring by means of questionnaires and collecting of spirometric data [28–34], remote consulting with the health center by means of the tool [26–29, 33], and distance learning [26, 27, 30, 32, 33, 35, 36]. Of note, there are studies that focus on a single sphere of content, while others include more than one. The study that we are presenting here includes several of these interventions.

In several studies telemedicine was used as a tool for controlling asthma and improving the quality of life of the users [26–36]. However, it is difficult to establish standards for the content of an application for asthma given the diversity of the interventions and the variability of the results obtained from them [37].

In the studies that we analyzed it was demonstrated that interventions that used a mobile application to provide information to participants regarding inhaled therapy and a personalized plan of action improved control of the illness and adherence to treatment [29, 32]. In contrast, in those studies not making using of a mobile application, but rather a website or SMS, the improvements were not significant, nor were there clinical benefits [34–36]. Another point to make note of is that applications that include reminders sent by SMS, both for taking medication and for completing questionnaires, showed a much higher level of compliance than did the interventions that did not include reminders [26, 34]. Nemanic T et al. achieved a participant compliance level of 80% with ACT and 96% with PEF thanks to SMS reminders [34].

In terms of communication between the patient and the professionals, the applications that allowed the patient to maintain direct contact with the healthcare professionals achieved improved asthma self-management [27, 29]. They also produced a reduction in daytime symptoms, number of hospitalizations, and visits to the emergency room.

Our intervention has sought to draw together elements from all the interventions that have achieved significant results and assemble them in a personalized application with which the patient will have available all the information needed to improve control over the illness, and through which the healthcare team can intervene in a much more efficient manner.

### Ethical considerations

The study is in compliance with the principles of the Helsinki Declaration, and it was approved by the Ethical Committee of the Bellvitge University Hospital (reference ICPS023/22). The personal data obtained will be confidential (Organic Law 3/2018, December 5, governing protection of personal data and the guarantee of digital rights) which supersedes Organic Law 15/1999, December 5, on the protection of personal data, and by extension, in the UE (General Regulation (UE) 2016/679 on the protection of data). Total confidentiality of the data obtained is guaranteed by the assignment of an identification number to each patient and by the creation of a database specific to this study in which data will be included in an encoded format.

All of the tests carried out by the patients in the present protocol are part of normal clinical practice in the treatment of patients with asthma. Therefore, participation in the study does not represent any additional risk to that associated with the normal care provided to asthmatic patients.

The data collected for this study will be encoded and only the principal investigator of the study and his collaborators will be able to associate the data with the medical history of the patients. Therefore, the identity of the patients will not be revealed to anyone except in the event of a medical emergency or legal requirement thereof.

Access to the personal information shall remain limited to the principal investigator of the study and his collaborators, as well as to the clinical research ethical committee when required to verify data and study procedures, at all times respecting the confidentiality of the data in accordance with the applicable legislation.

### Limitations

As this is a single-center study, the results may not be representative of the general population, which limits our ability to extrapolate the findings to different clinical settings. In an attempt to offset this limitation, and to ensure that the results are as extrapolable as possible, we will use the standards laid out in the Spanish Guide for the Management of Asthma (GEMA) [1]. To learn the degree of control of the illness we will take into account the number of exacerbations suffered in the past year, the asthma symptoms measured with ACT [19], and pulmonary function, using as measuring tools the PEF and the FEV1. With the aim of determining the degree of adherence to treatment we will use the TAI questionnaire and will review the electronic history of withdrawal from prescribed medication [1, 20]. For quality of life, we will make recourse to the AQLQ and the HAD [21, 22].

Given that the proposed intervention has simple masking, the research team and the clinical staff will be aware at all times which patients belong to which group, and this could influence the behavior of the staff as well as the manner in which the results are evaluated. As a result of randomization, it may be difficult to ensure that the groups are completely comparable in all areas.

Another limitation to bear in mind is the time stipulated for follow-up, which is limited and which may impede assessment of the effectiveness of the telemedicine program over the long-term in the control of asthma.

Finally, the participants may not comply completely with the recommendations made to them, or they may abandon the study prematurely, which would cause skewing in the results and render assessment of the real effectiveness of the telemedicine program in the control of asthma difficult. In addition, assessment of the control of asthma often involves subjective measurement of symptoms and quality of life, which could be influenced by the individual perception of the participants.

## Data Availability

No datasets were generated or analysed during the current study.
